# Supplementary material for: Games and enculturation: A cross-cultural analysis of cooperative goal structures in Austronesian games
Source: PLoS One. 2021 Nov 24;16(11):e0259746. doi: 10.1371/journal.pone.0259746 (PMC8612520; doi:10.1371/journal.pone.0259746)
Supplement: S1 File — (PDF) [file pone.0259746.s001.pdf]

Games and enculturation: A cross-cultural analysis of cooperative goal structures in Austronesian games

S1: Supporting Information

Sarah M. Leisterer-Peoples, Cody T. Ross, Simon J. Greenhill,
Susanne Hardecker, Daniel B. M. Haun

1 Game Counts

As mentioned in the main text, we use several filters provided by the AustroGames database [1] to filter the games. These filters were applied using the `game_filter( )` function from the AustroGames package [2] in R. The sample size of games after each of these steps is provided in Table 1. The final number of games of each goal structure type for each ethnolinguistic group is provided in Table 2. We used the following filters:

1. the description must have been coded as a game
2. games must have been linked to an Austronesian Basic Vocabulary Database code [ABVD, 3]
3. games must be described in enough detail to assign a goal structure code
4. games must not be of non-local origin
5. games must occur within cultural groups in the Austronesian language phylogeny [4]
6. the game descriptions must correspond to the same time frame as the covariate data from Pulotu, ±50 years
7. games must occur in cultural groups with covariate data in Pulotu [5]

Table 1: The filters applied to the AustroGames database [1] with the sample sizes after each step. The “Games remaining” column refers to the sample size of games after each step. The “Games dropped” column indicates the number of games that were removed from the sample by applying a respective filter to the results of the previous step. The “Groups remaining” column refers to the sample size of ethnolinguistic groups after each step. The abbreviation “MCCT” refers to the maximum clade credibility tree, and the abbreviation “ABVD” refers to the Austronesian basic vocabulary database code.

**Filter**	**Games remaining**	**Games dropped**	**Groups remaining**
(None)	952	-	-
Coded as a game	907	45	80
Linked to an ABVD	764	143	79
Coded goal structure	489	275	67
Excluding non-local origin	466	23	66
ABVD on the MCCT	430	36	55
Matching time frames with Pulotu ± 50 yrs	172	258	27
Covariate data in Pulotu	168	4	25
**Total**	**168**	-	**25**

The “Games dropped” column in Table 1 indicates that most games were dropped by using the “coded goal structure” filter (275 games dropped), the “matching time frames with Pulotu ±50 years” filter (258 games dropped), and the “linked to an ABVD” filter (143 games dropped). To our knowledge, the main difference between the games that were kept in the final dataset and those that were removed by using these filters lies in the quality of the game descriptions provided by the ethnographic materials. The game descriptions varied largely in their level of detail [1]. The most common reasons that some games could not be assigned a goal structure code were: 1) a lack of information about the rules of the game, 2) a lack of information about the players of the game, and 3)a lack of information about the general set-up of the game. Game descriptions that could not be linked with an ABVD code either did not contain sufficient information to assign an ABVD code, or the ABVD code could not be mapped on to the Austronesian language phylogeny [4]. Finally, game descriptions that were excluded from the final dataset by the filtering step: “matching time frames with Pulotu ±50 years”, were either described more than 50 years before or 50 years after the cultural attributes. As described in Leisterer-Peoples et al. [1, p.9], we believe that this filtering step is important to reduce the possibility that games from an early time point are not associated with cultural variables at a later time point (e.g., we would not want cultural variables from the 1700s to be linked with game data from the 1900s, especially if the cultural variables have since changed).

The ethnolinguistic groups that were excluded from the final sample did not systematically vary geographically from the ethnolinguistic groups that were included in the final sample (see Figure 1). Games were not systematically dropped based on their cultural attributes (for more information on the games that were excluded during filtering, see the main text, Table 1, and the R code on GitHub). There were some moderate differences between the cultural groups that were included versus excluded from the final sample (see Table 3 to Table 8).

Table 2: The number of games of each goal structure type in the analyses ($N = 168$). For the analyses, the AustroGames data were grouped into three categories (solitary, competitive, cooperative).

**Culture**	**Solitary**	**Competitive**	**Cooperative**
Iban	1	1	0
Yamdena	0	1	0
Marquesan	0	2	0
Hawaiian	1	12	2
TahitianModern	0	1	0
Rarotongan	0	1	0
Tikopia	0	3	0
Rennellese	4	17	9
Samoan	2	7	11
Tongan	3	7	6
Marshallese	0	4	2
Chuukese	0	2	3
Woleai	0	4	1
Nahavaq	0	3	0
Teop	3	1	2
Wogeo	0	0	1
Bwaidoga	2	2	4
Kilivila	1	2	3
Motu	0	2	4
Mekeo	0	0	1
Baree	5	3	7
MerinaMalagasy	1	3	1
KayanUmaJuman	0	3	0
SubanunSindangan	0	0	1
ItnegBinongan	0	4	2

ABVD codes were used to match the game data with the cultural attributes provided by Pulotu. As such, we could only investigate the relationship between games and cultural covariates after filtering out games that could be assigned an ABVD code.

Table 3: The number of cultural groups included in the final sample and excluded during filtering steps, and the levels of social stratification provided by Pulotu [5]. We note here that we may have lost more data from non-stratified groups than from stratified groups during filtering.

**Social stratification**	**Excluded**	**Included**
0	10	13
1	3	11
NA	5	1

Table 4: The number of cultural groups included in the final sample and excluded during filtering steps, and the levels of land-based hunting in groups provided by Pulotu [5]. We note here that we may have lost more data during filtering from groups with low levels of land-based hunting in groups than from groups with this variable.

**Land**	**Excluded**	**Included**
0	13	18
1	2	7
NA	3	0

Figure 1: The ethnolinguistic groups included in the final sample (i.e., the black dots), and in the original sample (i.e., the red dots; 3 are not visible due to missing coordinates). The "original" sample was only subset using the filters "coded as a game", "linked to an ABVD code", and "linked to a Pulotu code" (Table 1). The R code for this plot is available in the GitHub repository. For further information on the filters, see [1].

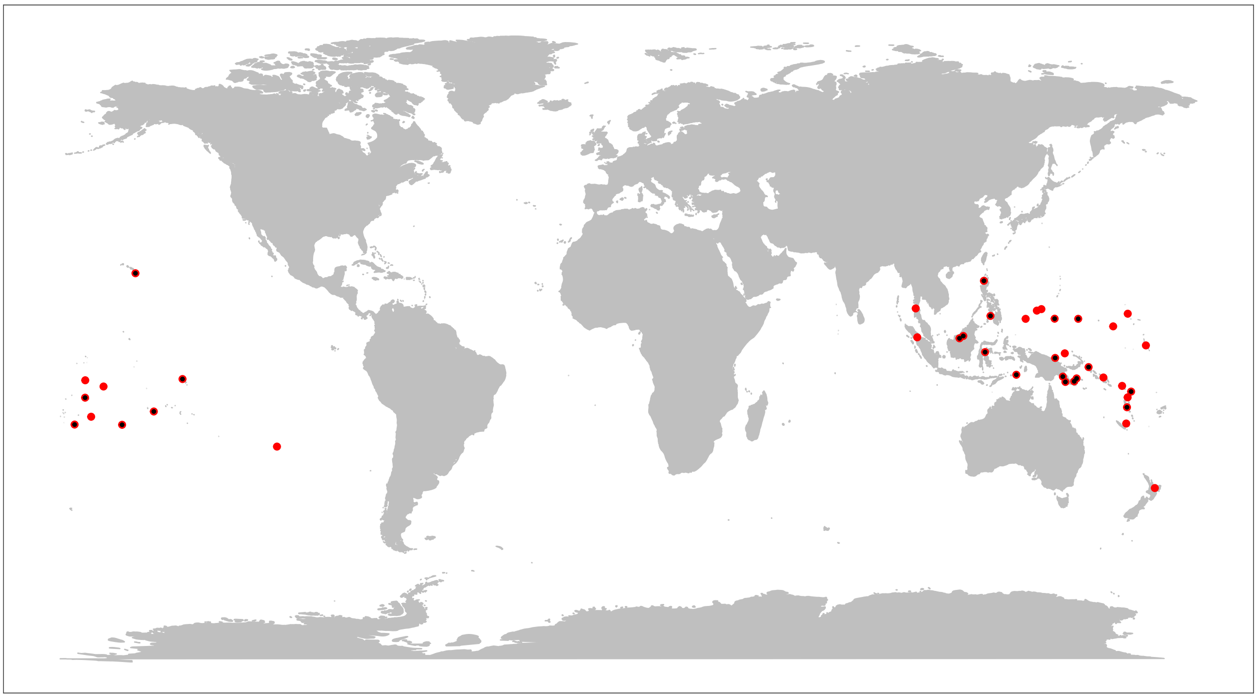

Figure 2: The number of games for each ethnolinguistic group included in the final sample ("final") and the number of games before the filtering steps ("orig") (see Figure 1 for more information). The dotted line marks 5 games. The colors represent the goal structures of the games. Please note that the y-axis differs for several plots.

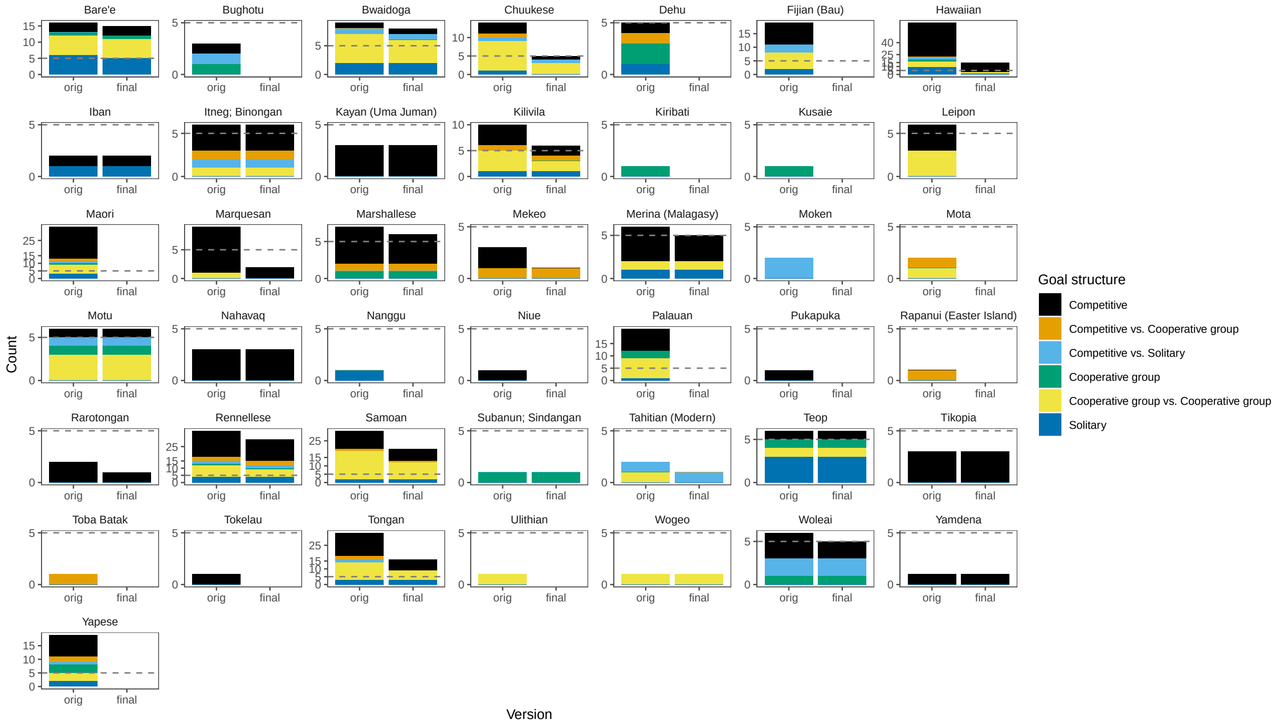

Table 5: The number of cultural groups included in the final sample and excluded during filtering steps, and the levels of water-based hunting in groups provided by Pulotu [5].

Water	Excluded	Included
0	9	9
1	6	16
NA	3	0

Table 6: The number of cultural groups included in the final sample and excluded during filtering steps, and the levels of intra-group conflict provided by Pulotu [5]. We note here that we may have lost more data during filtering from groups with intra-group conflict than from groups without intra-group conflict.

Intra-group conflict	Excluded	Included
0	3	13
1	11	12
NA	4	0

Table 7: The number of cultural groups included in the final sample and excluded during filtering steps, and the levels of intra-cultural conflict provided by Pulotu [5].

Intra-cultural conflict	Excluded	Included
0	6	7
1	11	18
NA	1	0

Table 8: The number of cultural groups included in the final sample and excluded during filtering steps, and the levels of inter-cultural conflict provided by Pulotu [5]. We note here that we may have lost more data during filtering from groups without inter-cultural conflict than from groups with inter-cultural conflict.

Inter-cultural conflict	Excluded	Included
0	14	12
1	3	13
NA	1	0

2 Statistical Methods

2.1 A basic model

The outcome of interest here is a count vector, Y, of the number of games of a given goal structure, s, observed in a given cultural group, c. Thus, let $Y_{[c]} \in \mathbb{N}^S$, where $S = 3$ is the number of categories of goal structures considered here. The total number of games, G, in cultural group c is then: $\sum_{s=1}^{S} Y_{[c][s]} = G_{[c]}$.

As such, we use a multinomial model for the outcomes:

$$Y_{[c]} \sim \text{Multinomial}(G_{[c]}, \text{Softmax}(\theta_{[c]})) \tag{1}$$

where:

$$\theta_{[c][s]} = \alpha_{[s]} + \beta_{[s]} Z_{[c]} + ... \tag{2}$$

$$\theta_{[c][S]} = 0 \tag{3}$$

Here, Equation 3 sets one category as a base case, and Equation 2 gives a linear model for the log odds of category s relative to the base case S. Each category s has its own regression equation, with a unique intercept, α, and slope, β. In the univariate models, we include only a single culture-level covariate, Z. In the multivariate models, we include two predictor variables and two slope parameters.

2.2 A phylogenetically controlled model

The previous model assumes that cultural groups are independent units. To control for the fact that our outcomes may be correlated due to shared phylogenetic history, we integrate correlated random effects into our model.

As before, we model outcomes as:

$$Y_{[c]} \sim \text{Multinomial}(G_{[c]}, \text{Softmax}(\theta_{[c]})) \tag{4}$$

but we now define:

$$\theta_{[c][s]} = \alpha_s + \beta_s Z_{[c]} + ... + \gamma_{[s][c]} \tag{5}$$

$$\theta_{[c][S]} = 0 \tag{6}$$

Assuming we have C cultural groups in our study, we use a vector of random effects, $\gamma_{[s]} \in \mathbb{R}^C$, for each category of game (except the base case). These random effects vectors are defined using a Gaussian process model:

$$\gamma_{[s]} \sim \text{Multivariate Normal Cholesky}((0, ..., 0)', \Sigma_{[s]}) \tag{7}$$

where:

$$\Sigma_{[s]} = \sigma_{[s]} L_{[s]} \tag{8}$$

and where $\sigma_{[s]}$ is a scale parameter and $L_{[s]}$ is a factor from the Cholesky decomposition of the correlation matrix $\rho_{[s]}$. To define $\rho_{[s]}$, we specify:

$$\rho_{[s][i,j]} = \phi_{[s]} \exp(-\psi_{[s]} D^2_{[i,j]}) \tag{9}$$

Here, $\phi_{[s]}$ is a maximal correlation parameter, $\psi_{[s]}$ is a decay parameter and, $D_{[i,j]}$ is the unit normalized phylogenetic distance between cultural groups i and j.

2.3 Priors

We use weakly regularizing priors on the intercept and slope parameters:

$$\alpha_{[s]} \sim \text{Normal}(0, 5) \tag{10}$$

$$\beta_{[s]} \sim \text{Normal}(0, 5) \tag{11}$$

The priors for phylogenetic control parameters are:

$$\phi_{[s]} \sim \text{Beta}(10, 2) \tag{12}$$

$$\psi_{[s]} \sim \text{Exponential}(2.5) \tag{13}$$

$$\sigma_{[s]} \sim \text{Exponential}(2.5) \tag{14}$$

2.4 Robustness checks

In the analyses presented in the main text, we used the maximum-clade credibility tree (MCCT). We conducted robustness checks using ten randomly selected trees from the Austronesian language phylogeny [4]. As indicated in Figure 3, the results do not differ from those in the main text using the MCCT.

Figure 3: The change in log relative frequency of each goal structure type as a function of predictor variables with phylogenetic controls included in the model. A positive parameter value corresponds to an increase in the frequency of goal structure type, while a negative parameter value corresponds to the opposite effect. Posterior densities show the results of pooling the densities for the same parameter in each of the 10 models with different phylogenetic trees. Vertical bars show 90% credible intervals.

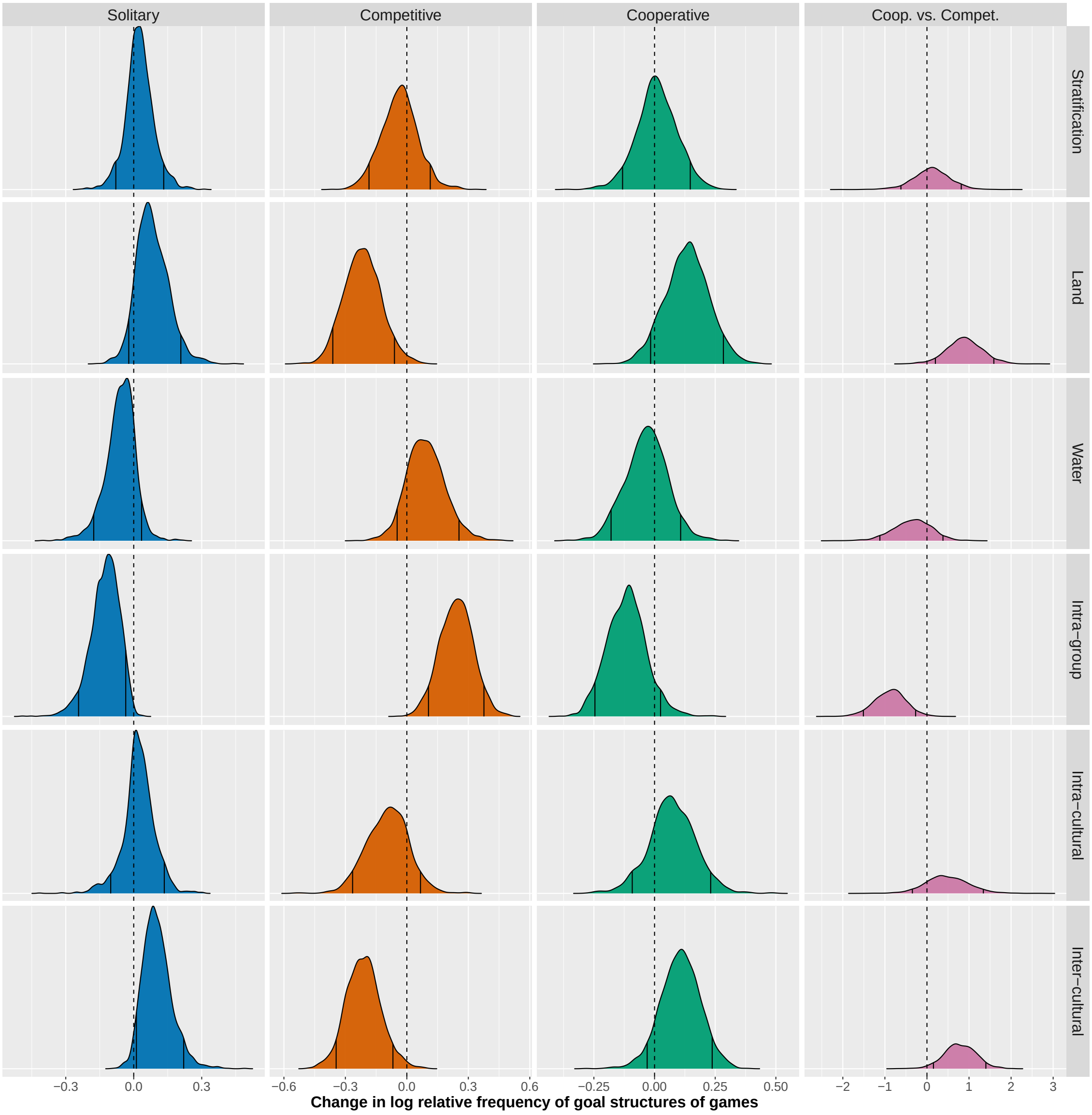

Figure 4: The change in log relative frequency of each goal structure as a function of predictor variables, with (basic, orange) and without phylogenetic controls (blue) included in the model. A positive parameter value corresponds to an increase in the frequency of goal structure types, while a negative parameter value corresponds to the opposite effect. Vertical bars show 90% credible intervals.

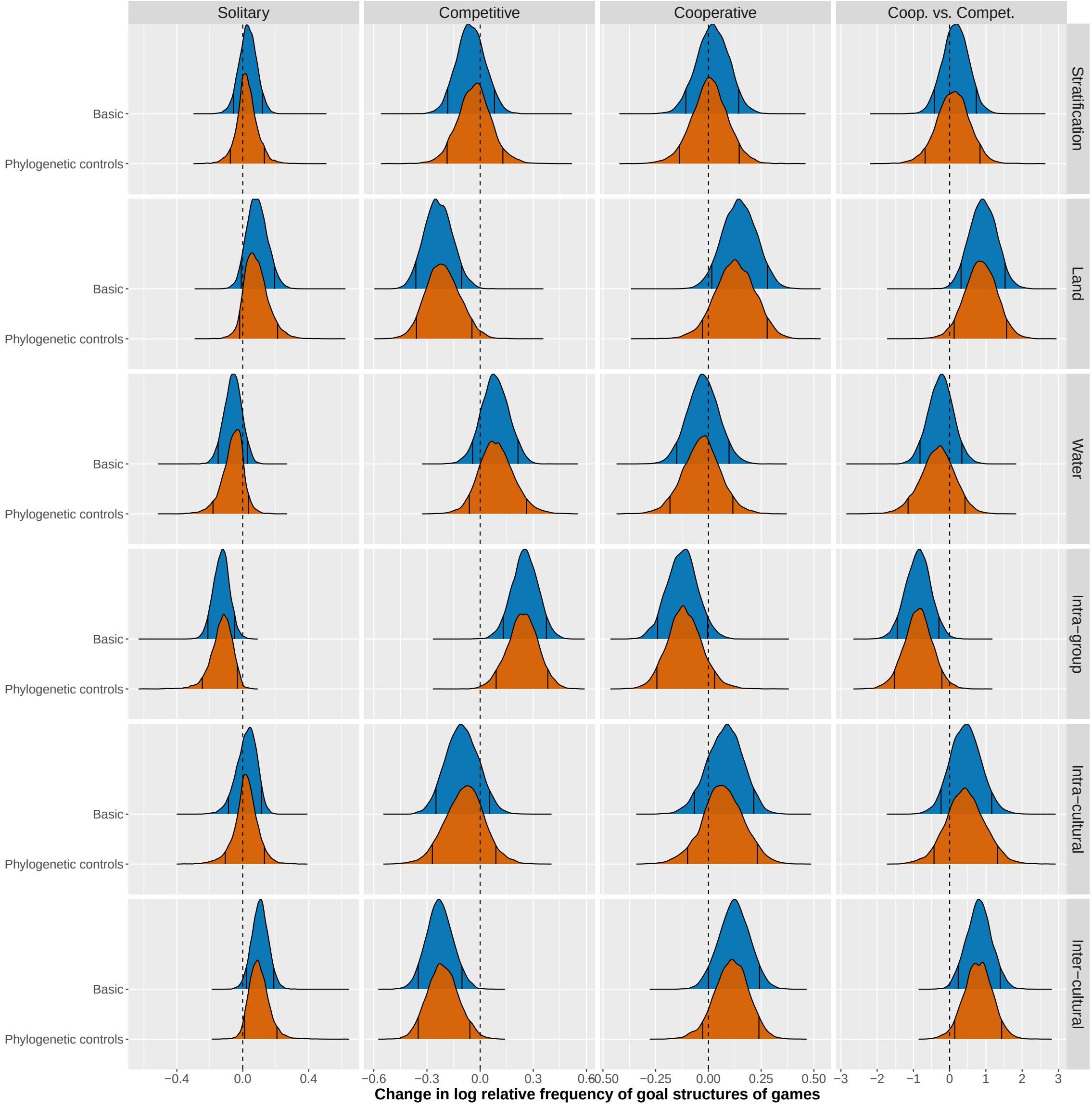

References

[1] Leisterer-Peoples SM, Hardecker S, Watts J, Greenhill SJ, Ross CT, Haun DB. The Austronesian Game Taxonomy: A cross-cultural dataset of historical games. Humanities & Social Sciences Communications. 2021;8(1):1–11. doi:10.1057/s41599-021-00785-y.

[2] Leisterer-Peoples SM, Hardecker S, Watts J, Greenhill SJ, Ross CT, Forkel R, et al.. The Austronesian Game Taxonomy: A cross-cultural dataset of historical games; 2021. Available from: https://zenodo.org/record/4675217.

[3] Greenhill SJ, Blust R, Gray RD. The Austronesian basic vocabulary database: from bioinformatics to lexomics. Evolutionary Bioinformatics. 2008;4:EBO–S893.

[4] Gray RD, Drummond AJ, Greenhill SJ. Language Phylogenies Reveal Expansion Pulses and Pauses in Pacific Settlement. Science. 2009;323(5913):479–483. doi:10.1126/science.1166858.

[5] Watts J, Sheehan O, Greenhill SJ, Gomes-Ng S, Atkinson QD, Bulbulia J, et al. Pulotu: database of Austronesian supernatural beliefs and practices. PloS one. 2015;10(9):e0136783.
