# Supplementary material for: Games and enculturation: A cross-cultural analysis of cooperative goal structures in Austronesian games
Source: PLoS One. 2021 Nov 24;16(11):e0259746. doi: 10.1371/journal.pone.0259746 (PMC8612520; doi:10.1371/journal.pone.0259746)
Supplement: Attachment — Submitted filename: Report-D-21-23199.pdf [file pone.0259746.s002.pdf]

# Games and enculturation: A cross-cultural analysis of games and values in Austronesia

## Summary

The authors employ a dataset on 168 historical games played by groups across the Austronesian language family and classify them on three categories: cooperative, competitive and solitary. The count of each game category within ethnolinguistic groups is explained with variables capturing (i) interdependence in foraging, (ii) conflict (intra- and inter- group), and (iii) social stratification. The authors find that land-based subsistence and a higher frequency of inter-group conflict are positively correlated with a higher count of games with cooperative goal structures, whereas the frequency of intra-group conflict is negatively related with the count of games with cooperative goal structures. Moreover, social stratification is not related to games with cooperative goal structures.

## Comment

This is an interesting article. In particular, I like the idea on how games serve as cultural training grounds. The discussion section is excellently written, so most of the potential concerns are adequately tackled beforehand.

I only have one comment: is there any gender division in the type of games that could be explored? If foraging and conflict activities are usually dominated by males, is it possible that any classification of gender-orientation (male-oriented, female-oriented, neutral) in the games can add any explanatory power?
