# Supplementary material for: Games and enculturation: A cross-cultural analysis of cooperative goal structures in Austronesian games
Source: PLoS One. 2021 Nov 24;16(11):e0259746. doi: 10.1371/journal.pone.0259746 (PMC8612520; doi:10.1371/journal.pone.0259746)
Supplement: Attachment — Submitted filename: Reponse_to_reviewers.pdf [file pone.0259746.s003.pdf]

Reviewer #1 wrote:

1. **The authors employ a dataset on 168 historical games played by groups across the Austronesian language family and classify them on three categories: cooperative, competitive and solitary. The count of each game category within ethnolinguistic groups is explained with variables capturing (i) interdependence in foraging, (ii) conflict (intra- and inter- group), and (iii) social stratification. The authors find that land-based subsistence and a higher frequency of inter-group conflict are positively correlated with a higher count of games with cooperative goal structures, whereas the frequency of intra-group conflict is negatively related with the count of games with cooperative goal structures. Moreover, social stratification is not related to games with cooperative goal structures.**

   Thank you very much for this comprehensive summary of our work.

2. **This is an interesting article. In particular, I like the idea on how games serve as cultural training grounds. The discussion section is excellently written, so most of the potential concerns are adequately tackled beforehand.**

   Thank you very much for your encouraging remarks.

3. **I only have one comment: is there any gender division in the type of games that could be explored? If foraging and conflict activities are usually dominated by males, is it possible that any classification of gender-orientation (male-oriented, female-oriented, neutral) in the games can add any explanatory power?**

   Thank you for this comment. We agree with reviewer # 1 that this is an interesting question and deserves future research. Unfortunately, the AustroGames database does not currently offer information on the sex of the players as this is under-described in the primary literature. The AustroGames dataset is a conglomerate of ethnographic and historical game descriptions, and as such, the amount of detail in these game descriptions varies to a large extent. The sex of the players was rarely mentioned in these game descriptions. Coding the sex of players would potentially require gathering further historical or ethnographic information on the games. We have added this suggestion from reviewer #1 in the manuscript as an impulse to future researchers:

   "Finally, future studies might also investigate whether gender-division plays a role in the distribution of games within cultures. Given that our cultural covariates are activities primarily performed by males, it might be that variation in the sex of game players across cultures has influenced the relationships uncovered here. Unfortunately, the AustroGames database does not currently offer information on the sex of the players as this is under-described in the primary literature. Future studies should focus on gathering further ethnographic materials that mention such details." (lines 438-445)

Reviewer #2 wrote:

1. **I should state that I am reviewing this manuscript from the perspective of a behavioral and experimental economist who has an interest in experiments using games. I will not be using the framework that a cultural anthropologist would bring to the paper, and so the authors and the editor should adjust their evaluation of my review accordingly.**
   I will make reference to some specific elements of the published criteria for PLOS-ONE acceptance as well as the PONE Reviewer Guidelines.

   Thank you for this background information. We value perspectives from outside our own discipline and are grateful for these comments.

2. **PONE reviewer guidelines ask "What are the main claims of the paper and how significant are they for the discipline?" This paper claims to show evidence that the distribution of games played within a family of small-scale societies is not random. They test for relationships among several factors potentially related to cultural evolutionary selection pressures, and find some evidence that three factors: land-based foraging interdependence, intra-group conflict, and inter-cultural conflict, are predictive of the number of cooperative and/or competitive games. Specifically, intra-cultural conflict and land foraging interdependence are associated with more cooperative games, while intra-group conflict is associated with more competitive games. Speaking from the perspective of 1) above, this is significant. (I also think it is quite interesting, but that is not a review criterion.) The field of anthropologically-informed cross-cultural comparisons has been undergoing a renaissance in recent years, and this paper is a contribution to that stream of research. Overall, if the authors can address the other concerns I have raised, I believe this will be a publishable MS.**

   Thank you for this encouraging summary of our work.

3. **PONE criteria for publication state "Results reported have not been published elsewhere." The material submitted constitutes a new analysis, and is a natural progression from the earlier publication by a number of the same authors of a description of the taxonomy of games in this family of cultures.**

   Thank you for this summary of our work.

4. **PONE reviewer guidelines ask "Is the manuscript well organized and written clearly enough to be accessible to non-specialists?" Here I have a couple of concerns that I suggest should be addressed before the MS is acceptable for publication. I start with two minor issues and progress to a more serious one. From the standpoint of (1) above, a minor point is that a technical term used in a manner specific to cultural anthropology is nowhere defined: "phylogenetic". (This could go in a footnote or the appendix, if the authors view it as too annoying to anthropological readers to put in the main text.)**

   Thank you for this comment. We have added more information on the term "phylogenetic" to the statistical analyses section of the manuscript:

   "Models without controls based on the Austronesian language phylogeny assume that each ethnolinguis-

tic group can be treated as an independent data point for the purpose of model fitting. However, these groups arguably share linguistic and cultural ancestry [21] that may have introduced correlations in outcomes. This shared ancestry is caused by the diversification of cultural groups over time whereby daughter cultures inherit many of the traits of their parent cultures before subsequently diverging themselves. Therefore, we use the Austronesian language phylogeny as a proxy for underlying cultural history [66, 74-78, e.g.]. Our models use the language phylogeny to introduce correlated random effects, which help to address Galton's Problem [79,80] and account for the potential non-independence of the ethnolinguistic groups in our study." (lines 286-295)

5. **The authors did not follow the submission guidelines for tables, which state "Tables are inserted immediately after the first paragraph in which they are cited." They put Table 1 of the main text in the Supplementary Materials, and completely omitted the key main text results table, Table 2. I was able to get a version of Table 2 by request to the editor, and what I got was not in publication-ready format, but an excel spreadsheet. So, first of all, the authors need to get the correct publication-formatted tables in the right place.**

   Thank you for this comment. We would like to apologize for this misunderstanding. The corresponding author used the PLOS ONE LaTeX template to write the manuscript. In this template, figures should be excluded from the manuscript. The corresponding author overgeneralized this to tables as well. We apologize for this and have include the tables in the correct positions in the manuscript. The tables are located between lines 204-205 and 369-370.

6. **But second, I was quite puzzled to see that Table 2 is in a format with each model in a row. Now I may be at cross purposes with the way anthropologists regularly do things, but this does not make sense to me. I suggest that the authors re-format so that each model is a column (this is certainly the standard for anything remotely econometric in flavor). This is much easier to understand, in my opinion. (Of course, if I really am at cross purposes to the regression table norm for anthropology, the authors are free to ignore this suggestion.)**

   We agree with the reviewer that there are different norms for how to present information in different scientific fields, but the information content of the table is equivalent whether we transpose the table. If the table were transposed, such that each model were its own column, we would have a very wide table with 19 columns and 6 rows, that will not fit within a standard-size PDF document. In our opinion, this presentation would be less accessible to the reader, so we keep the table in its current format.

7. **There is some kind of textual confusion in the MS, as a result of which it is just not clearly specified how the identified games are grouped for the statistical analysis. The statement about categories starting at line 196 on page 8/13 (starting with "Due to small sample sizes . . .") presumably was intended to spell this out. However, there is a period in the middle of what was perhaps intended to be a sentence, and the verb or verbs are missing. So, the authors just do not clearly explain themselves on this point. This needs to be repaired.**

   Thank you for this comment. We agree with reviewer #2—this sentence makes no sense in the initial manuscript. This was caused by a formatting mistake that we made in LaTeX. We have corrected this mistake in the current manuscript. This sentence should have read, and now reads, as:

"Due to small sample sizes in several of the goal structure categories (e.g., purely cooperative games; see Table 1 for sample sizes and Fig. 1 for details on the goal structures), we collapse the goal structures of games into three main groups: cooperative games *(i.e., all games with cooperative interactions: cooperative group, cooperative group versus cooperative group, competitive versus cooperative group),* competitive games *(i.e., games without cooperative interactions, but with competitive interactions: competitive versus solitary, competitive), and* solitary games *(i.e., games with neither cooperative nor competitive interactions: solitary)."* (lines 197-204)

8. **I have a final set of concerns that flow from PONE guidelines and standards. PONE Reviewer Guidelines state "Conclusions are presented in an appropriate fashion and are supported by the data." On this point the authors generally do a good job in the main text. However, they relapse back to statements that are a bit too unqualified in the conclusion. The evidence presented is suggestive, not conclusive. And it is consistent with the view that "games can play a functional role in human culture by mimicking overt real-world behavior, serving as cultural training grounds", but while this is currently our best conjecture at the relationship, it is not established by this paper. The abstract is considerably better in its wording.**

   Thank you for this comment. We agree with reviewer #2. We have edited the conclusion to remove the causal wording and make clear that the results are correlative.

   "Non-human animals engage in play(ful) behavior [109-114], but only humans play rule-based games [111]. Rule-based games are more than just child's play. Our study provides evidence for a relationship between the goal structure of games and the social and ecological environments of the ethnolinguistic groups that play them. The type and intensity of conflict, as well as the extent of interdependence in the acquisition of sustenance on land, appear to be correlated with the occurrence of specific types of game goal structures in our dataset. This evidence, though correlational, contributes to a growing body of literature which suggests that games may play a functional role in human culture by mimicking overt real-world behavior, thus serving as training grounds for norms and behaviors that are relevant for a particular socio-ecological context. However, future experimental studies with larger sample sizes would be needed to verify such predictions." (lines 558-569)

9. **The PONE submission guidelines state: "Include details of any corrections applied to account for multiple comparisons. If corrections were not applied, include a justification for not doing so". I believe I am correct in saying the authors have missed the ball on this one. I should be clear that in this kind of exploratory research using unique cultural/historical data, it is not feasible to collect new samples from the same population, and so multiple comparisons are appropriate to explore the patterns in the data. The authors need to acknowledge, however, that the statistical significance levels have to be taken with a certain grain of salt, because of data limitations.**

   Thank you for your comment. Reviewer #2 expresses two concerns here: one is limitations of our data (i.e., sample size), the other is statistical significance levels.

   We agree with reviewer #2 that there are limitations to our data. We acknowledge the small sample size throughout the paper and recommend future research on lines 197, 424-425, 497-500, 536-537, 566-568.

Regarding the second concern, we do not frame our paper as using a null-hypothesis rejection procedure. We understand that controlling for multiple testing and adjusting statistical significance levels is common practice in frequentist statistical procedures. However, as is common in Bayesian statistical procedures, which we conduct here, we provide the credible intervals around the parameter estimates, but refrain from mentioning statistical significance throughout the paper. We have added details about the Bayesian approach to the statistical analysis section to clarify this for readers:

"We use univariate and multivariate, multinomial regressions using a Bayesian framework, coded in `Stan` [72], with and without phylogenetic controls, to estimate the relationship between the frequency of game goal structures and the presence or absence of cultural variables." (lines 274-277)

And we added information to the manuscript pertaining to multiple testing:

"We use weakly regularizing priors to prevent overfitting of the sample, but we do not apply corrections with regard to multiple testing, as we are using a Bayesian analysis framework, as opposed to a null-hypothesis rejection framework [73, see]." (lines 280-283)

Additionally, we have removed the term "significant" from our manuscript to remove any possible confusion this term may cause readers. (lines 53, 150, 215, 218, 220, 227)

Finally, we propose a relationship between the goal structure of games and cultural attributes as one possible explanation for the non-random distribution of games around the world. We frame this as an exploratory test, not as a definitive relationship, as reviewer #2 has also pointed out—our results are correlative, not causal. Future research is required, and we recommend several possible avenues to test this relationship in future research.

10. **The authors mention the sample size and potential bias issues that flow from the limitations of their data (page 18, line 409 and following lines on page 19.) The selection process is described starting on page 8, line 184, and following lines on page 9. What is missing are the statistics needed to see how the pruning of the initially broad list of games and societies was undertaken, along with any more specific comments about the potential for bias. For instance, in what order were criteria applied, and how many potential cases were lost due to each criterion? Additionally, I would expect to see some kind of statement as to what can be said about dropped cases, to the extent this is possible. For instance, do they vary from included cases in terms of any observable variables? I will speculate that not a lot can be said, and if so, perhaps this account will make up a paragraph or two in the supplemental materials that is referred to in the main text, but in my view something needs to be added. This is an exploratory work examining the relationship of multiple potential evolutionarily relevant causal factors to a specific type of outcome variable, but the lessons from narrower policy evaluation work are still relevant: it is always important to look out for selection effects that may be confounded with treatment effects. With the current paper's data there will be limited ability to do this, and the authors have noted the issue, but I suggest little more attention should be paid to what can be said about this issue from the data.**

Thank you for this comment. We agree with reviewer #2. We have added further information about the sample sizes after each filtering step to the supporting information file (see lines 1-34, table 1, figure 1-3 in the S1 File). We refer to this in the manuscript as follows:

"Additional information on the sample sizes after each filtering step, and the biases that could potentially be introduced by these filters, can be found in the S1 file." (lines 195-196)

The S1 file on this section reads:

The "Games dropped" column in Table 1 indicates that most games were dropped by using the "coded goal structure" filter (275 games dropped), the "matching time frames with Pulotu ±50 years" filter (258 games dropped), and the "linked to an ABVD" filter (143 games dropped). To our knowledge, the main difference between the games that were kept in the final dataset and those that were removed by using these filters lies in the quality of the game descriptions provided by the ethnographic materials. The game descriptions varied largely in their level of detail [1]. The most common reasons that some games could not be assigned a goal structure code were: 1) a lack of information about the rules of the game, 2) a lack of information about the players of the game, and 3)a lack of information about the general set-up of the game. Game descriptions that could not be linked with an ABVD code either did not contain sufficient information to assign an ABVD code, or the ABVD code could not be mapped on to the Austronesian language phylogeny [4]. Finally, game descriptions that were excluded from the final dataset by the filtering step: "matching time frames with Pulotu ±50 years", were either described more than 50 years before or 50 years after the cultural attributes. As described in Leisterer-Peoples et al. [1, p.9], we believe that this filtering step is important to reduce the possibility that games from an early time point are not associated with cultural variables at a later time point (e.g., we would not want cultural variables from the 1700s to be linked with game data from the 1900s, especially if the cultural variables have since changed). (lines 14-29)

Additionally, we examined the potential relationship between the cultural covariates and the games that were kept in the final sample and the games that were dropped during filtering. We do find some relationship between a few cultural covariates and the games that were filtered in/out of the final sample. As this examination was quite detailed, we make a broad statement in the S1 File and provide the R code for interested readers on GitHub. The S1 File reads as follows:

"The ethnolinguistic groups that were excluded from the final sample did not systematically vary geographically from the ethnolinguistic groups that were included in the final sample (see Figure 1). Games were not systematically dropped based on their cultural attributes (for more information on the games that were excluded during filtering, see the main text, Table 1, and the R code on GitHub). There were some moderate differences between the cultural groups that were included versus excluded from the final sample (see Table 3 to Table 8). ABVD codes were used to match the game data with the cultural attributes provided by Pulotu. As such, we could only investigate the relationship between games and cultural covariates after filtering out games that could be assigned an ABVD code." (lines 30-37)
